# CDKN2BAS polymorphisms are associated with coronary heart disease risk a Han Chinese population

**DOI:** 10.18632/oncotarget.12575

**Published:** 2016-10-11

**Authors:** Qingbin Zhao, Shudan Liao, Huiyi Wei, Dandan Liu, Jingjie Li, Xiyang Zhang, Mengdan Yan, Tianbo Jin

**Affiliations:** ^1^ Department of Geratology, The First Affiliated Hospital of Xi'an Jiaotong University, Xi'an, Shaanxi, China; ^2^ Department of Cardiology, Xi'an Center Hospital, Xi'an, Shaanxi, China; ^3^ Department of Endocrinology, The First Affiliated Hospital of Xi'an Jiaotong University, Xi'an, Shaanxi, China; ^4^ Key Laboratory of Resource Biology and Biotechnology in Western China (Northwest University), Ministry of Education, Xi'an, Shaanxi, China; ^5^ Xi'an Tiangen Precision Medical Institute, Xi'an, Shaanxi, China

**Keywords:** coronary heart disease, single nucleotide polymorphisms, CDKN2BAS, case-control, Gerotarget

## Abstract

The goal of our study was to determine whether *CDKN2BAS* polymorphisms are associated with coronary heart disease (CHD) risk in a Han Chinese population. Eight SNPs were genotyped in 676 men and 465 women. We used χ^2^ tests and genetic model analyses to evaluate associations between the SNPs and CHD risk. We found that rs10757274 was associated with an increased risk of CHD in both men (allele G: Odds ratio [OR] = 1.30, 95% confidence interval [CI]: 1.05-1.61, *P* = 0.018; codominant model: *P* = 0.042; recessive model: OR = 1.70, 95% CI: 1.10-2.62, *P* = 0.016; log-additive model: OR = 1.34, 95% CI: 1.05-1.71, *P* = 0.019) and women (dominant model: OR = 2.26, 95% CI: 1.28-3.99, *P* = 0.004). In addition, rs7865618 was associated with an 8.10-fold increased risk of CHD in women under a recessive model (OR = 8.10, 95% CI: 1.74-37.68, *P* = 0.006). Interestingly, the haplotype AA (rs10757274 and rs1333042) of *CDKN2BAS* was associated with decreased the risk of CHD in men (OR = 0.72, 95% CI: 0.55 - 0.95, *P* = 0.022).

## INTRODUCTION

Coronary heart disease (CHD), is a major cause of morbidity and mortality worldwide [1] and one of the most common chronic inflammatory diseases, characterized by remodeling and narrowing of the blood vessels (coronary arteries) that supply oxygen and blood to the heart [2]. CHD is a complex disease influenced by both environmental and genetic factors [3, 4]. Epidemiological studies have identified many risk factors for CHD, including age, gender, smoking, obesity, diabetes, hyperlipidemia, hypertension, lack of exercise, and dietary factors. Twin and family studies have demonstrated that a significant proportion (40-50%) of susceptibility to CHD is inherited [5]. Although atherosclerosis is one of the main pathophysiological mechanisms of CHD [2], the responsible molecular and genetic determinants remain largely unidentified. Recently, both genome-wide association studies (GWAS) and candidate gene studies have reported that *CDKN2BAS* (cyclin-dependent kinase inhibitor 2B antisense RNA) is a risk gene for CHD susceptibility [6–8].

*CDKN2BAS* encodes an antisense non-coding RNA, and is located near the *CDKN2A*-*CDKN2B* gene. The precise function of *CDKN2BAS* is unclear, but it regulates the expression of neighboring protein-coding genes, like *CDKN2A*, *CDKN2B*, and *MTAP*, that enhance the progression of atherosclerosis by influencing vascular remodeling, thrombogenesis, and plaque stability [9, 10]. Therefore, *CDKN2BAS* expression plays a pivotal role in the development of CHD by altering the dynamics of vascular cell proliferation. In addition, single nucleotide polymorphisms (SNPs) in *CDKN2BAS* are associated with the risk of multiple diseases, including comprising CHD [7, 9, 11], myocardial infarction [7], type 2 diabetes [12], ischemic stroke [13], and periodontitis [14].

While many studies have demonstrated that polymorphisms in *CDKN2BAS*are associated with the risk of CHD, few studies have focused on the effects of these alterations on susceptibility to CHD in the Han Chinese population. Therefore, we performed a case-control study to investigate the associations between these SNPs and the risk of CHD in the Han Chinese men and women.

## RESULTS

Basic information on the eight SNPs in *CDKN2BAS*, including chromosomal position, role, allele, minor allele frequency (MAF), and Hardy-Weinberg Equilibrium (HWE) test results are shown in Table 1. All SNPs were in HWE in the control groups (*P* > 0.05). We then compared the differences in frequency distributions of alleles between cases and controls using Chi-squared tests and found that the frequency of allele “G” of rs10757274 in *CDKN2BAS* was higher in cases, and it was associated with a 1.30-fold increased risk of CHD in men at a 5% level (OR = 1.30, 95% CI: 1.05-1.61, *P* = 0.018).

**Table 1:** Allele frequencies in cases and controls and odds ratio estimates for CHD

SNP-ID	Gene	Chr	Band	Position	Role	Alleles (A/B)	Men						Women					
							MAF		HWE	OR	95%CI	*P*	MAF		HWE	OR	95%CI	*P*
							Case	Control					Case	Control				
rs7865618	CDKN2BAS	9	9p21.3	22031005	Intron	G/A	0.116	0.132	0.827	0.86	0.62-1.19	0.352	0.139	0.105	1.000	1.38	0.92-2.07	0.119
rs11790231	CDKN2BAS	9	9p21.3	22053591	Intron	A/G	0.148	0.140	0.672	1.06	0.78-1.45	0.689	0.148	0.163	0.527	0.89	0.62-1.30	0.553
rs1412832	CDKN2BAS	9	9p21.3	22077543	Intron	C/T	0.259	0.285	0.619	0.87	0.69-1.12	0.279	0.273	0.285	1.000	0.94	0.70-1.27	0.690
rs6475606	CDKN2BAS	9	9p21.3	22081850	Intron	C/T	0.275	0.303	0.470	0.87	0.69-1.10	0.254	0.303	0.297	0.581	1.03	0.77-1.38	0.839
rs1333040	CDKN2BAS	9	9p21.3	22083404	Intron	C/T	0.273	0.294	0.538	0.90	0.71-1.15	0.408	0.294	0.293	0.677	1.00	0.75-1.35	0.985
rs1537370	CDKN2BAS	9	9p21.3	22084310	Intron	C/T	0.273	0.299	0.331	0.88	0.69-1.12	0.305	0.303	0.297	0.581	1.03	0.77-1.38	0.839
rs10757274	CDKN2BAS	9	9p21.3	22096055	Intron	G/A	0.483	0.418	1.000	1.30	1.05-1.61	0.018	0.458	0.425	0.555	1.14	0.87-1.50	0.338
rs1333042	CDKN2BAS	9	9p21.3	22103813	Intron	A/G	0.310	0.353	0.574	0.83	0.66-1.04	0.104	0.324	0.340	0.898	0.93	0.70-1.24	0.626

Chr: Chromosome; MAF: Minor allele frequency; HWE: Hardy-Weinberg equilibrium; OR: Odds ratio; 95% CI: 95% Confidence interval.

*P* values were calculated from Chi-square /Fisher's exact.

*P* <0.05 indicates statistical significance.

We further assessed the association between each SNP and CHD risk using unconditional logistic regression analysis after adjusting for age. The results of genetic model analyses (codominant, dominant, recessive, log-additive) are presented in Table 2. We identified that rs10757274 was associated with increased CHD risk in men under a codominant model (*P* = 0.042); a recessive model (GG *vs* AA+AG: OR = 1.70, 95% CI: 1.10-2.62, *P* = 0.016) and a log-additive model (OR = 1.34, 95% CI: 1.05-1.71, *P* = 0.019). Interestingly, rs10757274 was also associated with increased CHD risk in women under both a codominant model (*P* = 0.004) and a dominant model (AG+GG *vs* AA: OR = 2.26, 95% CI: 1.28-3.99, *P* = 0.004). Allele “G” of rs7865618 was also associated with increased CHD risk under a codominant model (*P* = 0.021). A similar trend was observed for rs7865618, which was associated with a 6.79-fold increased risk of CHD in women under a recessive model (GG *vs* AA+GA: OR = 8.10, 95% CI: 1.74-37.68, *P* = 0.006).

**Table 2:** Genetic models analyses of the association between *CDKN2BAS* SNPs and CHD susceptibility in men and women

SNP-ID	Model	Genotype		Men				Women		
			Case	Control	OR (95% CI)	P-value	Case	Control	OR (95% CI)	P-value
rs7865618	Codominant	A/A	227 (78.3%)	290 (75.3%)	1		127 (77%)	240 (80%)	1	0.021
		G/A	59 (20.3%)	88 (22.9%)	0.80 (0.52-1.23)	0.430	30 (18.2%)	57 (19%)	1.07 (0.57-1.98)	
		G/G	4 (1.4%)	7 (1.8%)	0.54 (0.13-2.18)		8 (4.8%)	3 (1%)	8.20 (1.76-38.35)	
	Dominant	A/A	227 (78.3%)	290 (75.3%)	1		127 (77%)	240 (80%)	1	
		G/A-G/G	63 (21.7%)	95 (24.7%)	0.78 (0.52-1.18)	0.240	38 (23%)	60 (20%)	1.37 (0.77-2.45)	0.290
	Recessive	A/A-G/A	286 (98.6%)	378 (98.2%)	1		157 (95.2%)	297 (99%)	1	
		G/G	4 (1.4%)	7 (1.8%)	0.57 (0.14-2.28)	0.420	8 (4.8%)	3 (1%)	8.10 (1.74-37.68)	0.006
	Log-additive	---	---	---	0.78 (0.54-1.14)	0.200	---	---	1.58 (0.97-2.58)	0.068
rs11790231	Codominant	G/G	217 (74.6%)	281 (73.6%)	1		121 (73.3%)	208 (69.3%)	1	0.520
		A/G	62 (21.3%)	95 (24.9%)	0.99 (0.65-1.50)	0.380	39 (23.6%)	86 (28.7%)	0.74 (0.42-1.29)	
		A/A	12 (4.1%)	6 (1.6%)	2.15 (0.71-6.52)		5 (3%)	6 (2%)	1.23 (0.28-5.36)	
	Dominant	G/G	217 (74.6%)	281 (73.6%)	1		121 (73.3%)	208 (69.3%)	1	
		A/G-A/A	74 (25.4%)	101 (26.4%)	1.07 (0.72-1.60)	0.720	44 (26.7%)	92 (30.7%)	0.78 (0.45-1.33)	0.350
	Recessive	G/G-A/G	279 (95.9%)	376 (98.4%)	1		160 (97%)	294 (98%)	1	
		A/A	12 (4.1%)	6 (1.6%)	2.15 (0.71-6.51)	0.160	5 (3%)	6 (2%)	1.33 (0.31-5.76)	0.700
	Log-additive	---	---	---	1.14 (0.81-1.60)	0.450	---	---	0.85 (0.53-1.35)	0.490
rs1412832	Codominant	T/T	161 (55.5%)	194 (50.5%)	1		84 (50.9%)	153 (51%)	1	0.760
		T/C	108 (37.2%)	161 (41.9%)	0.76 (0.52-1.09)	0.250	72 (43.6%)	123 (41%)	1.02 (0.62-1.69)	
		C/C	21 (7.2%)	29 (7.5%)	0.70 (0.36-1.37)		9 (5.5%)	24 (8%)	0.71 (0.27-1.90)	
	Dominant	T/T	161 (55.5%)	194 (50.5%)	1		84 (50.9%)	153 (51%)	1	
		T/C-C/C	129 (44.5%)	190 (49.5%)	0.75 (0.53-1.06)	0.100	81 (49.1%)	147 (49%)	0.97 (0.60-1.57)	0.890
	Recessive	T/T-T/C	269 (92.8%)	355 (92.5%)	1		156 (94.5%)	276 (92%)	1	
		C/C	21 (7.2%)	29 (7.5%)	0.79 (0.42-1.51)	0.480	9 (5.5%)	24 (8%)	0.70 (0.27-1.83)	0.470
	Log-additive	---	---	---	0.80 (0.61-1.05)	0.110	---	---	0.92 (0.63-1.36)	0.680
rs6475606	Codominant	T/T	156 (53.6%)	183 (47.7%)	1		77 (46.7%)	146 (48.7%)	1	0.920
		C/T	110 (37.8%)	169 (44%)	0.73 (0.50-1.05)	0.210	76 (46.1%)	130 (43.3%)	0.91 (0.55-1.50)	
		C/C	25 (8.6%)	32 (8.3%)	0.76 (0.40-1.43)		12 (7.3%)	24 (8%)	0.89 (0.36-2.23)	
	Dominant	T/T	156 (53.6%)	183 (47.7%)	1		77 (46.7%)	146 (48.7%)	1	
		C/T-C/C	135 (46.4%)	201 (52.3%)	0.73 (0.52-1.04)	0.077	88 (53.3%)	154 (51.3%)	0.90 (0.56-1.47)	0.680
	Recessive	T/T-C/T	266 (91.4%)	352 (91.7%)	1		153 (92.7%)	276 (92%)	1	
		C/C	25 (8.6%)	32 (8.3%)	0.88 (0.48-1.61)	0.670	12 (7.3%)	24 (8%)	0.94 (0.39-2.27)	0.880
	Log-additive	---	---	---	0.81 (0.62-1.06)	0.120	---	---	0.93 (0.63-1.36)	0.700
rs1333040	Codominant	T/T	156 (53.6%)	188 (49.1%)	1		80 (48.5%)	148 (49.3%)	1	0.930
		T/C	111 (38.1%)	165 (43.1%)	0.78 (0.54-1.12)	0.380	73 (44.2%)	128 (42.7%)	0.91 (0.55-1.52)	
		C/C	24 (8.2%)	30 (7.8%)	0.81 (0.43-1.55)		12 (7.3%)	24 (8%)	0.90 (0.36-2.24)	
	Dominant	T/T	156 (53.6%)	188 (49.1%)	1		80 (48.5%)	148 (49.3%)	1	
		T/C-C/C	135 (46.4%)	195 (50.9%)	0.78 (0.55-1.11)	0.170	85 (51.5%)	152 (50.7%)	0.91 (0.56-1.48)	0.710
	Recessive	T/T-T/C	267 (91.8%)	353 (92.2%)	1		153 (92.7%)	276 (92%)	1	
		C/C	24 (8.2%)	30 (7.8%)	0.91 (0.49-1.69)	0.760	12 (7.3%)	24 (8%)	0.94 (0.39-2.27)	0.880
	Log-additive	---	---	---	0.85 (0.65-1.11)	0.230	---	---	0.93 (0.64-1.37)	0.720
rs1537370	Codominant	T/T	156 (53.6%)	185 (48%)	1		77 (46.7%)	146 (48.7%)	1	0.920
		C/T	111 (38.1%)	170 (44.2%)	0.73 (0.51-1.06)	0.240	76 (46.1%)	130 (43.3%)	0.91 (0.55-1.50)	
		C/C	24 (8.2%)	30 (7.8%)	0.79 (0.41-1.51)		12 (7.3%)	24 (8%)	0.89 (0.36-2.23)	
	Dominant	T/T	156 (53.6%)	185 (48%)	1		77 (46.7%)	146 (48.7%)	1	
		C/T-C/C	135 (46.4%)	200 (52%)	0.74 (0.53-1.05)	0.093	88 (53.3%)	154 (51.3%)	0.90 (0.56-1.47)	0.680
	Recessive	T/T-C/T	267 (91.8%)	355 (92.2%)	1		153 (92.7%)	276 (92%)	1	
		C/C	24 (8.2%)	30 (7.8%)	0.91 (0.49-1.70)	0.760	12 (7.3%)	24 (8%)	0.94 (0.39-2.27)	0.880
	Log-additive	---	---	---	0.82 (0.62-1.07)	0.150	---	---	0.93 (0.63-1.36)	0.700
rs10757274	Codominant	A/A	82 (28.2%)	130 (33.8%)	1		39 (23.6%)	102 (34%)	1	0.004
		A/G	137 (47.1%)	188 (48.8%)	1.16 (0.78-1.72)	0.042	101 (61.2%)	141 (47%)	2.60 (1.44-4.71)	
		G/G	72 (24.7%)	67 (17.4%)	1.86 (1.13-3.05)		25 (15.2%)	57 (19%)	1.52 (0.72-3.23)	
	Dominant	A/A	82 (28.2%)	130 (33.8%)	1		39 (23.6%)	102 (34%)	1	
		A/G-G/G	209 (71.8%)	255 (66.2%)	1.33 (0.91-1.93)	0.130	126 (76.4%)	198 (66%)	2.26 (1.28-3.99)	0.004
	Recessive	A/A-A/G	219 (75.3%)	318 (82.6%)	1		140 (84.8%)	243 (81%)	1	
		G/G	72 (24.7%)	67 (17.4%)	1.70 (1.10-2.62)	0.016	25 (15.2%)	57 (19%)	0.81 (0.43-1.51)	0.500
	Log-additive	---	---	---	1.34 (1.05-1.71)	0.019	---	---	1.31 (0.92-1.87)	0.130
rs1333042	Codominant	G/G	139 (47.9%)	162 (42.6%)	1		74 (44.9%)	130 (43.3%)	1	0.810
		G/A	122 (42.1%)	168 (44.2%)	0.88 (0.61-1.27)	0.120	75 (45.5%)	136 (45.3%)	0.87 (0.52-1.45)	
		A/A	29 (10%)	50 (13.2%)	0.55 (0.31-0.97)		16 (9.7%)	34 (11.3%)	0.81 (0.36-1.84)	
	Dominant	G/G	139 (47.9%)	162 (42.6%)	1		74 (44.9%)	130 (43.3%)	1	
		G/A-A/A	151 (52.1%)	218 (57.4%)	0.79 (0.56-1.12)	0.190	91 (55.1%)	170 (56.7%)	0.86 (0.53-1.39)	0.530
	Recessive	G/G-G/A	261 (90%)	330 (86.8%)	1		149 (90.3%)	266 (88.7%)	1	
		A/A	29 (10%)	50 (13.2%)	0.59 (0.34-1.01)	0.050	16 (9.7%)	34 (11.3%)	0.87 (0.40-1.90)	0.730
	Log-additive	---	---	---	0.78 (0.61-1.01)	0.058	---	---	0.89 (0.62-1.28)	0.530

OR: Odd ratio; 95% CI: 95% Confidence.

*P* values were calculated from the Wald test.

*P* < 0.05 indicates statistical significance.

**Figure 1:**
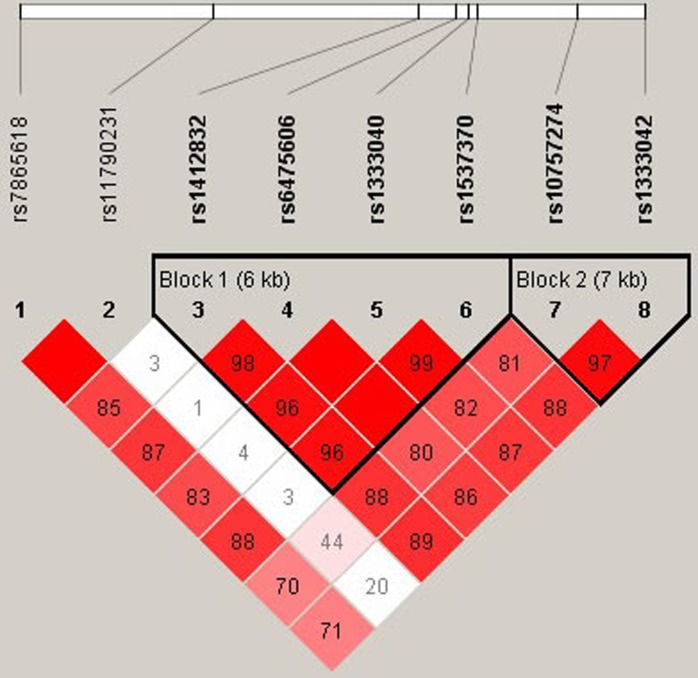
Haplotype block map for the eight SNPs in *CDKN2BAS* in men Block 1 includes rs1412832, rs6475606, rs1333040 and rs1537370; Block 2 includes rs10757274 and rs1333042. The LD between two SNPs is standardized D'.

Linkage Disequilibrium (LD) analysis demonstrated that two SNPs (rs10757274 and rs1333042) in *CDKN2BAS* showed strong linkage (Figure 1). Furthermore, the haplotype AA (rs10757274 and rs1333042) was significantly associated with a decreased risk of CHD in men (OR = 0.72, 95% CI: 0.55 - 0.95, *P* = 0.022; Table 3).

**Table 3:** Haplotype frequencies and association with the risk of CHD in men

Chr	Gene	SNPs	Haplotype	F_A	F_U	OR (95% CI)	*P*-value
9	CDKN2BAS	rs10757274|rs1333042	GG	0.481	0.415	1	---
			AA	0.309	0.350	0.72 (0.55 - 0.95)	0.022
			AG	0.208	0.232	0.75 (0.54 - 1.04)	0.083

F_A: Frequency in cases; F_U: Frequency in controls; OR: Odd ratio; 95% CI: 95% Confidence.

*P* values were calculated using the Wald test.

*P* < 0.05 indicates statistical significance.

Additionally, we also investigated the associations between *CDKN2BAS* polymorphisms and CHD-related biochemical indicators, including triglyceride (TG), total cholesterol (TC), high-density lipoprotein cholesterol (HDL-C), low density lipoprotein cholesterol (LDL-C), creatinine (CRE), neutrophil (NEU), platelet distribution width (PDW) as shown in Table 4. The levels of HLD-C with rs6475606 (*P* = 0.022) and rs1333040 (*P* = 0.045) in men, and rs1412832 (*P* = 0.017) in women, were different between groups. Levels of CRE with rs7865618 (*P* = 0.035) and rs1412832 (*P* = 0.015) in men, and rs11790231 and rs6475606 (*P* = 0.033) in women, were also different between groups. In addition, the levels of NEU with three SNPs (rs7865618: *P* = 0.037, rs11790231: *P* = 0.018 and rs6475606: *P* = 0.027) and the levels PDW with four SNPs (rs7865618: *P* = 0.029, rs1333040: *P* = 0.047, rs1537370: *P* = 0.020 and rs10757274: *P* = 0.047) were different between groups, but only in women.

**Table 4:** Lipid levels according to genotype in men and women

Sex	SNP-ID	Genotype (N)	TG		TC		HDL-C		LDL-C		CRE		NEU		PDW	
			Mean ± SD	*P*	Mean ± SD	*P*	Mean ± SD	*P*	Mean ± SD	*P*	Mean ± SD	*P*	Mean ± SD	*P*	Mean ± SD	*P*
Men	rs7865618	CC(137)	1.70±0.11		3.90±0.09		1.20±0.02		1.84±0.08		86.82±1.75		5.24±0.27		14.54±0.25	
		TT(19)	1.74±0.27	0.524	3.95±0.43	0.607	1.15±0.07	0.549	1.87±0.24	0.927	85.63±3.18	0.035	5.18±0.69	0.699	14.16±0.59	0.496
		CT(119)	1.93±0.19		4.04±0.11		1.09±0.02		1.88±0.07		80.58±1.72		5.56±0.29		14.11±0.27	
	rs11790231	GG(4)	1.54±0.46		3.54±0.77		1.00±0.01		1.75±0.52		74.63±9.56		4.67±0.63		13.03±1.11	
		AA(217)	1.77±0.12	0.752	3.96±0.08	0.745	1.10±0.02	0.644	1.87±0.06	0.953	84.50±1.38	0.546	5.22±0.20	0.210	14.22±0.20	0.277
		GA(53)	1.95±0.20		4.00±0.16		1.08±0.04		1.84±0.09		82.90±2.22		6.02±0.52		14.82±0.39	
	rs1412832	GG(203)	1.79±0.12		3.95±0.08		1.10±0.02		1.86±0.06		83.94±1.33		5.57±0.23		14.41±0.20	
		AA(12)	1.46±0.15	0.659	3.80±0.26	0.803	0.99±0.06	0.281	1.88±0.20	0.994	99.43±11.89	0.015	5.42±0.81	0.161	12.67±0.74	0.186
		AG(60)	1.92±0.17		4.03±0.17		1.10±0.03		1.86±0.11		81.30±1.59		4.68±0.33		14.28±0.36	
	rs6475606	CC(20)	1.76±0.24		3.64±0.25		0.95±0.04		1.72±0.13		79.66±4.68		6.02±1.05		14.00±0.61	
		TC(101)	1.78±0.15	0.979	4.05±0.13	0.371	1.12±0.03	0.022	1.91±0.08	0.651	84.30±2.00	0.613	5.76±0.30	0.131	14.11±0.28	0.519
		TT(153)	1.82±0.15		3.95±0.09		1.10±0.02		1.85±0.08		84.36±1.52		5.05±0.24		14.50±0.24	
	rs1333040	CC(24)	1.77±0.20		3.78±0.22		0.98±0.05		1.79±0.13		86.96±6.80		5.97±0.90		13.51±0.59	
		TT(147)	1.79±0.15	0.992	3.94±0.09	0.638	1.11±0.02	0.045	1.86±0.08	0.907	84.74±1.55	0.501	5.12±0.25	0.320	14.61±0.25	0.148
		CT(104)	1.81±0.15		4.02±0.13		1.11±0.03		1.88±0.08		82.46±1.63		5.59±0.29		14.09±0.26	
	rs1537370	CC(23)	1.64±0.16		3.68±0.21		0.98±0.05		1.79±0.13		86.34±7.09		6.05±0.93		13.43±0.62	
		TC(105)	1.84±0.15	0.867	4.04±0.13	0.410	1.11±0.03	0.055	1.88±0.08	0.915	82.62±1.62	0.592	5.57±0.29	0.299	14.10±0.25	0.134
		TT(147)	1.79±0.15		3.94±0.09		1.11±0.02		1.86±0.08		84.74±1.55		5.12±0.25		14.61±0.25	
	rs10757274	CC(23)	1.64±0.16		3.68±0.21		0.98±0.05		1.79±0.13		86.34±7.09		6.05±0.93		13.43±0.62	
		TT(147)	1.79±0.15	0.867	3.94±0.09	0.410	1.11±0.02	0.055	1.86±0.08	0.915	84.74±1.55	0.592	5.12±0.25	0.299	14.61±0.25	0.134
		CT(105)	1.84±0.15		4.04±0.13		1.11±0.03		1.88±0.08		82.62±1.62		5.57±0.29		14.10±0.25	
	rs1333042	GG(69)	1.74±0.15		3.95±0.13		1.15±0.03		1.94±0.15		84.05±1.95		4.85±0.36		14.49±0.35	
		AA(78)	1.69±0.16	0.650	3.88±0.15	0.766	1.07±0.03	0.098	1.81±0.09	0.644	82.23±2.22	0.591	5.71±0.40	0.258	14.24±0.31	0.856
		AG(128)	1.90±0.17		4.01±0.10		1.08±0.02		1.84±0.06		85.10±1.84		5.43±0.26		14.28±0.26	
Women	rs7865618	CC(78)	1.88±0.17		4.33±0.11		1.16±0.03		1.96±0.07		72.77±1.89		4.01±0.24		13.77±0.36	
		TT(14)	2.09±0.42	0.320	4.78±0.30	0.213	1.24±0.07	0.264	2.24±0.23	0.383	70.62±4.72	0.849	5.94±1.02	0.037	16.28±0.82	0.029
		CT(62)	1.62±0.09		4.23±0.14		1.22±0.03		2.05±0.10		70.82±3.47		4.32±0.34		13.97±0.41	
	rs11790231	GG(7)	1.41±0.13		4.49±0.28		1.12±0.10		2.15±0.12		89.85±25.84		6.83±0.87		14.08±1.01	
		AA(120)	1.72±0.12	0.119	4.24±0.10	0.163	1.19±0.02	0.759	1.99±0.07	0.615	71.83±1.53	0.033	4.14±0.24	0.018	14.09±0.31	0.986
		GA(28)	2.22±0.26		4.66±0.22		1.20±0.05		2.12±0.14		66.94±1.65		4.28±0.38		13.98±0.57	
	rs1412832	GG(113)	1.84±0.13		4.35±0.11		1.16±0.02		2.02±0.07		71.43±2.13		4.44±0.25		13.69±0.31	
		AA(5)	1.59±0.13	0.769	5.26±0.42	0.082	1.39±0.12	0.017	2.70±0.35	0.079	67.38±4.81	0.788	4.27±0.99	0.490	14.90±1.40	0.051
		AG(37)	1.69±0.15		4.15±0.13		1.26±0.04		1.94±0.11		73.57±3.07		3.86±0.35		15.13±0.49	
	rs6475606	CC(8)	2.22±0.44		5.00±0.21		1.27±0.08		2.45±0.21		89.42±22.61		6.44±0.98		14.23±0.92	
		TC(68)	1.91±0.20	0.291	4.33±0.13	0.179	1.19±0.03	0.681	1.99±0.08	0.212	69.15±1.62	0.033	3.97±0.28	0.027	13.38±0.37	0.065
		TT(79)	1.65±0.09		4.26±0.12		1.19±0.03		2.01±0.08		72.22±1.96		4.35±0.30		14.63±0.38	
	rs1333040	CC(11)	1.99±0.34		4.82±0.19		1.23±0.07		2.33±0.17		82.56±17.23		5.93±0.82		14.18±0.80	
		TT(73)	1.62±0.09	0.276	4.25±0.13	0.248	1.19±0.03	0.838	2.00±0.09	0.326	72.20±2.13	0.172	4.28±0.31	0.068	14.73±0.40	0.047
		CT(71)	1.94±0.20		4.34±0.12		1.18±0.03		1.19±0.08		69.74±1.50		4.05±0.28		13.39±0.36	
	rs1537370	CC(11)	1.99±0.34		4.82±0.19		1.23±0.07		2.33±0.17		82.56±17.23		5.93±0.82		14.18±0.80	
		TC(69)	1.95±0.20	0.263	4.36±0.12	0.221	1.19±0.03	0.850	2.00±0.08	0.327	69.40±1.55	0.152	3.96±0.27	0.050	13.27±0.36	0.020
		TT(75)	1.62±0.09		4.23±0.13		1.19±0.03		2.00±0.09		72.42±2.06		4.36±0.31		14.78±0.39	
	rs10757274	CC(11)	1.99±0.34		4.82±0.19		1.23±0.07		2.33±0.17		82.56±17.23		5.93±0.82		14.18±0.80	
		TT(73)	1.62±0.09	0.276	4.25±0.13	0.248	1.19±0.03	0.838	2.00±0.09	0.326	72.20±2.13	0.172	4.28±0.31	0.068	14.73±0.40	0.047
		CT(71)	1.94±0.20		4.34±0.12		1.18±0.03		1.19±0.08		69.74±1.50		4.05±0.28		13.39±0.36	
	rs1333042	GG(24)	1.62±0.18		3.95±0.23		1.24±0.06		1.83±0.16		70.55±3.08		4.13±0.55		15.27±0.67	
		AA(36)	1.99±0.21	0.528	4.52±0.14	0.116	1.17±0.03	0.554	2.16±0.11	0.223	74.91±5.50	0.608	4.68±0.44	0.584	13.45±0.60	0.113
		AG(95)	1.77±0.14		4.36±0.11		1.19±0.03		2.02±0.07		70.93±1.70		4.20±0.26		14.03±0.31	

TG: Triglyceride; TC: Total cholesterol; HDL-C: High-density lipoprotein cholesterol; LDL-C: Low density lipoprotein cholesterol; CRE: Creatinine; NEU: Neutrophil;

PDW: Platelet distribution width

## DISCUSSION

The goal of our case-control study was to explore the associations of eight polymorphisms in *CDKN2BAS* with the risk of CHD in a Han Chinese population. We found that rs10757274 was associated with an increased risk of CHD both in men and women. However, rs7865618 was correlated with an increased risk of CHD only in women. In addition, we also found that the haplotype “AA” (rs10757274 and rs1333042) of *CDKN2BAS* was associated with a decreased CHD risk in men.

Previous studies have demonstrated that *CDKN2BAS* transcript levels show a strong correlation with the severity of atherosclerosis. In addition, the modulation of *CDKN2BAS* expression influences CHD susceptibility. The function of *CDKN2BAS* is unknown, but it regulates the expression of *CDKN2A* and *CDKN2B*, which encode cyclin-dependent kinase inhibitors 2A and 2B, indicating a regulatory role for *CDKN2BAS* in cellular proliferation. Polymorphisms at the 9p21 region may induce higher expression of the *CDKN2BAS* transcript, thereby inhibiting the expression of *CDKN2A* and *CDKN2B*.

Several studies have demonstrated a strong association of rs10757274 with CHD in Pakistani [9], Caucasian [15], and South-West Iranian [16] population. Our results are consistent with these previous findings d. However, one previous study found no association of rs10757274 with CHD in a Han Chinese population (Shenzhen) [17].The differences between our study and this study are likely due to differences in the two study populations. How rs10757274 affects the risk of CHD is unclear, but this SNP may regulate the expression of *EU741058* and *p16INK4a*, which modulate the risk of developing CHD [18]. Some studies have also suggested that rs10757274impairs the mechanical properties of the arterial wall and thus influences vascular diseases [19].

It was previously shown that rs1333042 is associated with the risk of CHD in the Han Chinese [20, 21] and Saudi populations [7], which is inconsistent with our findings. The differences in these studies may be explained by ethnic differences, environment, or lifestyle that also affected the development of CHD. We also observed that rs7865618 was associated with CHD risk in women. However, this SNP has previously only been found to interact with other SNPs to affect the development of CHD [6]. In future studies, we will verify our results using a larger sample size.

We didn't observe any association between rs1333040 and the risk of CHD, which is consistent with the studies by Cao et al. [21] and Golabgir Khademi et al. [22]. This SNP was significantly associated with the risk of CHD in a Northern Indian population [23] and in African American women [24]. Genetic variation and differences in life styles among populations probably explain the population disparities in the association of this SNP and susceptibility to CHD. Furthermore, recent studies have reported that the levels of TG, TC, HDL-C, LDL-C, CRE, NEU and PDW effectively predict the risk of CHD [25–28]. Interestingly, we also demonstrated that SNPs in *CDKN2BAS* were correlated with the levels of these biochemical indicators and differed between cases and controls.

Several limitations should be acknowledged in the present study. First, the sample size was relatively small and the participants were limited to Chinese ethnicity. Second, there were differences in some clinical characteristics between the patients and controls. Although several confounders have been adjusted for the statistical analyses, we could not completely eliminate the potential influences of these factors on the results. Finally, the biological mechanism of genetic variants in *CDKN2BAS* was not investigated in this study. It will be important to follow up and validate our findings with larger sample sizes.

In conclusion, our results suggest that, in a Chinese Han population, rs10757274 in *CDKN2BAS* is associated with the risk of CHD both in men and women, rs7865618 is correlated with an increased risk of CHD only in women, and the haplotype AA (rs10757274 and rs1333042) of *CDKN2BAS* is associated with a decreased CHD risk in men. Thus, these SNPs could have clinical importance as pre-diagnostic markers. Further study is required to determine the functional effects of these SNPs and validate these findings in larger populations.

## MATERIALS AND METHODS

### Ethics statement

Written informed consent was obtained from all study participants before the interview. This study protocol was approved by the Ethical Committee of the Yanan University Affiliated Hospital and the First Affiliated Hospital of Xi'an Jiaotong University, and complied with the World Medical Association Declaration of Helsinki.

### Study participants

The study included 676 men (291 CHD cases with a mean age of 60 years and 385 healthy controls with a mean age of 48 years) and 465 women (165 CHD cases with a mean age of 64 years and 300 healthy controls with a mean age of 50 years). All CHD cases were recruited from the Cardiovascular Internal Medicine Department of Yanan University Affiliated Hospital between February 2014 and April 2015. The 685 healthy controls were randomly selected from physical examination center of the same hospital during the same period. The inclusion and exclusion criteria for participants were as follows: First, all subjects were of the ethnic Han origin and not related to each other. Second, all participants diagnosis we based on standardized electrocardiogram, echocardiography, blood tests and coronary angiography and judged by two or three independent cardiologists. Third, all individuals were excluded from the study if they had other cardiac diseases (congenital heart disease, cardiomyopathy, or rheumatic heart disease), diabetes, hypertension, or severe liver or kidney disease.s, Patients who had previously received angioplasty, intravenous thrombolysis, coronary artery stents, or coronary artery bypass surgery were also excluded. Basic characteristics of all enrolled controls were collected with a standard epidemiological questionnaires conducted by well-trained interviewers. The cases information was collected through consultation with treating physicians or from medical chart review. Peripheral venous blood (5 ml) was collected from each participant using vacutainer tubes containing ethylene diamine tetra-acetic acid (EDTA) and then stored at -80°C. A clinical examination at which a blood sample was drawn for routine analysis of blood levels, biochemical tests, coagulation function, and genetic analyses.

### SNP selection and genotyping

The eight SNPs in *CDKN2BAS* (rs7865618, rs1179023, rs1412832, rs6475606, rs1333040, rs1537370, rs10757274 and rs1333042) were selected from previous reports for their association with CHD [9, 21, 23, 29, 30]. The minor allele frequency of each SNP was > 5% in the HapMap of the Chinese Han Beijing (CHB) population. Genomic DNA was extracted from whole blood using the GoldMag-Mini Whole Blood Genomic DNA Purification Kit according to the manufacturer's protocol (GoldMag. Co. Ltd., Xi'an, China). DNA concentration and purity were evaluated using a spectrophotometer (NanoDrop 2000; Thermo Fisher Scientific, Waltham, MA, USA). Polymerase chain reaction (PCR) and extension primers for the SNPs were designed using the Sequenom MassARRAY Assay Design 3.0 software (Sequenom, San Diego, CaliforniaCA, USA). Genotyping was performed using the Sequenom MassARRAY platform (Sequenom, San Diego, CA, USA) according to the standard instructions. Sequenom Typer 4.0 software was used for data management and analyses.

### Statistical analysis

SPSS 19.0 (SPSS Inc., Chicago, IL, USA) and Microsoft Excel (Microsoft Corp., Redmond, WA, USA) were used for statistical analyses. Genotypic frequencies in controls (men and women) were tested for departure from HWE using a Fisher's exact test. The allelic frequencies were compared between cases and controls by Chi-squared test/Fisher's exact test, and the relative risk was estimated by odd ratios (ORs) and 95% confidence intervals (CIs). The genetic model analyses (codominant, dominant, recessive, log-additive) were applied using PLINK software to assess the significance of SNPs. ORs and 95% CIs were calculated using unconditional logistic regression analysis with adjustment for age. The *p* values were calculated with the Wald test. Haploview software (version 4.2) was used for analyses of the pairwise linkage disequilibrium (LD), haplotype structure and genetic association at polymorphism loci. All *p* values were two-sided, and *p* < 0.05 is considered statistically significant.
